# Can DNA help trace the local trade of pangolins? Conservation genetics of white-bellied pangolins from the Dahomey Gap (West Africa)

**DOI:** 10.1186/s12862-022-01971-5

**Published:** 2022-02-14

**Authors:** Stanislas Zanvo, Chabi A. M. S. Djagoun, Akomian F. Azihou, Bruno Djossa, Komlan Afiademanyo, Ayodeji Olayemi, Clément Agbangla, Brice Sinsin, Philippe Gaubert

**Affiliations:** 1 Laboratory of Applied Ecology, Faculty of Agronomic Sciences, University of Abomey-Calavi, 01 BP 526, Cotonou, Benin; 2 Laboratoire Evolution et Diversité Biologique (EDB), CNRS/UPS/IRD, Université Toulouse III Paul Sabatier – Bâtiment 4R1, 118 route de Narbonne, 31062 Toulouse cedex 9, France; 3 Laboratoire de Foresterie et de Conservation des Bioressources (LaFCBio), Ecole de Foresterie Tropicale, Université Nationale d’Agriculture, Kétou, Benin; 4 Laboratoire de Génétique Moléculaire et d’Analyse des Génomes, Faculté des Sciences et Techniques, Université d’Abomey-Calavi, 01BP 526, Cotonou, Bénin; 5 Département de Zoologie et de Biologie Animale, Université de Lomé, BP 1515, Lomé, Togo; 6 Natural History Museum, Obafemi Awolowo University, HO, Ile-Ife, 220005 Nigeria

**Keywords:** Microsatellites, Conservation genetics, Demographic decline, Trade tracing, White-bellied pangolin, Dahomey Gap

## Abstract

**Background:**

African pangolins are currently experiencing unprecedented levels of harvesting, feeding both local demands and the illegal international trade. So far, the lack of knowledge on the population genetics of African pangolins has hampered any attempts at assessing their demographic status and tracing their trade at the local scale. We conducted a pioneer study on the genetic tracing of the African pangolin trade in the Dahomey Gap (DG). We sequenced and genotyped 189 white-bellied pangolins from 18 forests and 12 wildlife markets using one mitochondrial fragment and 20 microsatellite loci.

**Results:**

Tree-based assignment procedure showed that the pangolin trade is endemic to the DG region, as it was strictly fed by the the Dahomey Gap lineage (DGL). DGL populations were characterized by low levels of genetic diversity, an overall absence of equilibrium, important inbreeding levels, and lack of geographic structure. We identified a 92–98% decline in DGL effective population size 200–500 ya—concomitant with major political transformations along the ‘Slave Coast’—leading to contemporaneous estimates being inferior to minimum viable population size (< 500). Genetic tracing suggested that wildlife markets from the DG sourced pangolins through the entire DGL range. Our loci provided the necessary power to distinguish among all the genotyped pangolins, tracing the dispatch of a same individual on the markets and within local communities. We developed an approach combining rarefaction analysis of private allele frequencies with cross-validation of observed data that traced five traded pangolins to their forest origin, c. 200–300 km away from the markets.

**Conclusions:**

Although the genetic toolkit that we designed from traditional markers can prove helpful to trace the illegal trade in pangolins, our tracing ability was limited by the lack of population structure within the DGL. Given the deleterious combination of genetic, demographic, and trade-related factors affecting DGL populations, the conservation status of white-bellied pangolins in the DG should be urgently re-evaluated.

**Supplementary Information:**

The online version contains supplementary material available at 10.1186/s12862-022-01971-5.

## Background

Pangolins (Order Pholidota) are considered the most trafficked wild mammals in the world, with c. 900,000 individuals seized over the last 20 years [1]. Although pangolins have been—mistakingly—highlighted as potential intermediary hosts of the COVID-19 pandemia [2], the volumes traded have remained unsustainably high [3]. As the exponential demand from the Traditional Chinese Medicine (TCM) market has reduced the populations of Asian pangolins, new trafficking routes have emerged from Africa [4]. Between 2015 and 2019, an estimated > 400,000 African pangolins were seized en route to Asian markets [1, 5]. Consequently, African pangolins are currently experiencing unprecedented levels of harvesting, for both local demands and the illegal international trade, with a possible influence of the Chinese diaspora on the African trade networks and dynamics [4, 6, 7].

The white-bellied pangolin (WBP; *Phataginus tricuspis*) is the species with the largest range in tropical Africa and is the pangolin most frequently sold in the African bushmeat markets [5]. It is also the most highly represented African species in international seizures of pangolin scales [4, 8]. Recent genetic investigations have shown that WBP consisted of six cryptic, geographically traceable lineages [9], one of which occurs ‘outside’ the rainforest blocks, in a West African savannah corridor interspersed with highly fragmented forest cover, the Dahomey Gap [10]. The Dahomey Gap lineage (DGL) is of particular importance as it is endemic to a unique biogeographical zone in western Africa (from Togo to Benin and southwestern Nigeria [9]) and likely is the only pangolin species surviving in the Dahomey Gap [11, 12].

DGL populations currently suffer from intense levels of deforestation and hunting. Populations are fragmented into generally small patches of forest islands, and have drastically decreased in abundance through the last decades [11, 12]. In Togo and Benin, WBP is hunted for its meat and use in traditional medicine, both contributing to its overexploitation [11, 12]. With recent seizures of scales intended for the international trade (Cotonou airport; [4]), a prominent proportion of DGL in Asian seizures [8], and the established trafficking hub in neighbouring Nigeria [13], there is a serious risk that DGL populations are exploited at unsustainable levels.

The genetic toolkit has been successfully applied to trace the illegal wildlife trade, by providing accurate information on the species traded and their geographic origins [14, 15]. Recently, the genetic distinctiveness among the different WBP lineages has been used to assign the regional origins of international seizures of pangolin scales [8]. However, the lack of knowledge on the population genetics of WBP has hampered any attempts at assessing their demographic status and tracing their local—e.g., country-scale—trade (see [16]). The stakes behind tracing the local WBP trade are high, as they reside in identifying the source populations feeding the market (and thus the market network), but also estimating the number of individuals traded (e.g., from scales on stalls and in seizures), and, therefore, better informing conservation actions to mitigate this trade. We propose a pioneering investigation on the utility of the genetic toolkit applied to the conservation genetics of WBP, based on recently developed microsatellite markers [17] in combination with a mitochondrial marker used to delineate among WBP lineages [9]. Our general objective is to provide a detailed overview of the genetic status of DGL populations and their traceability on the local pangolin trade. Our specific objectives encompass the assessment of (i) population structure and diversity within the DGL, (ii) the demographic history of this endemic lineage, and (iii) the resolutive power of our genetic markers for tracing the pangolin trade in the Dahomey Gap.

## Methods

### Genetic sampling and wet laboratory procedures

We collected a total of 189 WBP samples across Benin (6° 10′–11° 00′ N), Togo (8° 10–9° 0′) and southwestern Nigeria (6° 10′–11° 00′ N). Our sampling effort included all of the species occurrence zones in Benin [11], five geographically proximate forests in Togo (111 samples from 18 forests), nine major traditional medicine markets (TMMs; 71) from Benin and Togo, and three bushmeat markets in Benin and Nigeria (7). Samples from forests (reference samples) and wildlife markets (individuals to be traced) were collected simultaneously from April 2018 to February 2020. For the reference samples, collection was done with local hunters from the villages surrounding the occurrence habitats of pangolins using a snowball technique [11]. Sample types varied from fresh tissue, skin and tongue (59), to dried skin and tissue (60), and scale connective tissue (70) (see Additional file 1: Table S1). Thirty-six samples were taken from carcasses having received preservartive chemical treatments [7]. Free consent from local hunters and market sellers was obtained before collecting samples. We relied on an opportunistic sampling strategy [18], without financial incentives. The samples collected from the forest were traced to their original location after information was provided by hunters [11].

DNA extraction from fresh and scale connective tissues was performed using the NucleoSpin® Tissue Kit (Macherey–Nagel, Hoerdt, France), following manufacturer’s recommendations. The final elution step was repeated twice in 50 µl Elution buffer to increase DNA yield. The samples treated with chemicals were extracted following a modified CTAB protocole including upstream TE washing baths and Dithiothreitol (DTT [19]). Elution volumes varied from 30 to 100 µl nuclease free water, depending on the size of the DNA pellet. DNA concentrations were estimated on the NanoDrop 1000 Spectrophotometer (ThermoFisher Scientific, Illkirch-Graffenstaden, France).

We amplified a mitochondrial fragment of 432 bp from Control Region 1 (CR1) following Gaubert et al. [9]. PCR products were sequenced at Genoscreen (https://www.genoscreen.fr/en; Lille, France) and Macrogen Europe (https://dna.macrogen-europe.com/en; Amsterdam, the Netherlands). Sequences were aligned manually with BioEdit v7.0.5 [20] and the unique haplotypes were submitted to Genbank under accession numbers OK275650–OK275662.

We amplified 20 microsatellite markers developed from the genome of WBP in four multiplexes following Aguillon et al. [17]. PCR triplicates were conducted for dried and chemically treated samples to mitigate the potential issue of allelic dropout and false alleles [21]. In our case, a consensus was considered met when at least two out of the three replicates indicated the presence of an allele. PCR products were separated on an automated sequencer at Genoscreen and GeT-PlaGe (https://get.genotoul.fr/; INRAE, Toulouse, France).

### Data analysis—control region 1

#### Clustering

We assessed the ‘endemicity’ of the pangolins from Togo, Benin and Nigeria (N = 168) through a distance-tree analysis including all the CR1 sequences of WBP available in Genbank (N = 100). All the sequences were aligned by-eye with BioEdit v7.0.5 [20]. Phylogenetic tree reconstruction was performed in MEGA-X v10.2.2 [22] using Neightbor Joining, 1,000 bootstrap replicates, Kimura 2-parameter model [23] and Gamma distribution (G). A pangolin was considered endemic to the DGL if its sequence clustered within the Dahomey Gap lineage as defined by [9].

#### Genetic diversity and structure

Genetic diversity and structure in DGL were estimated from sequences without missing data (N = 126). We used DnaSP v6.12 [24] to compute haplotype number (*h*), haplotype diversity (*Hd*) and nucleotide diversity (*π*) (Additional file 2: Table S2) for the six WBP lineages. We mapped the distribution of haplotypes in DGL using ArcGIS 10.1 (Esri France). We used Network v10.2.0.0 to build a median-joining haplotype network with ε = 0 to minimize alternative median networks.

#### Demographic history

Mismatch analysis was performed in Arlequin 3.5.2 [25] to test for signatures of demographic and spatial expansion in DGL, by calculating the sum of squared deviations (SSD) between observed and expected distributions using 1000 boostrap replicates [26].

We also tested for deviation from neutrality by computing a series of statistics in DnaSP, including Tajima’s D [27], Fu’s Fs [28], Harpending raggedness index *r* [29] and Ramos-Onsins and Rozas’ R2 [30]. We ran 1,000 replicates assuming a coalescent process with a neutral, infinite-sites model and large constant population sizes [31], to calculate the P-value of each observed statistics.

### Data analysis—microsatellites

#### Genetic diversity

Genious 9.0.5 [32] was used for allele scoring and genotype extraction through the Microsatellites plugin (https://www.geneious.com/features/microsatellite-genotyping/). Only the DGL individuals with at least 75% of genotyping success were considered for the analyses (N = 169). This arbitrary threshold coincides with ≥ 15 microsatellite markers, tripling the minimum number of loci needed to discriminate among individuals in the white-bellied pangolin lineage from western central Africa [17]. Genetic diversity at each locus was characterized through (i) number of alleles (Na), observed (*Ho*) and expected (*He*) heterozysity as computed from GenAlEx 6.5 [33], (ii) allelic richness (A_R_) and *F*_*IS*_ as estimated from FSTAT 2.9.4 [34]. Deviation from Hardy–Weinberg Equilibrum (HWE) was calculated for each locus in GenAlEx. Linkage Deseliquilibrum (LD) for all the pairs of loci was tested in FSTAT using 1000 randomisations with a Bonferroni correction. Null allele detection, assuming population at equilibrium, was done with Microcheker 2.2.3 [35] using a Bonferroni correction.

#### Genetic structure

Global genetic variance within the DGL was visualized through a Principal Coordinates Analysis (PCoA), using pairwise population matrix unbiased genetic distances in GenAlEx.

Pairwise differentiation (*F*_*ST*_) among forest populations (N = 104) was computed in Arlequin 3.5 [36] using three partition schemes (Additional file 2: Fig. S3): (i) a 6-partition scheme considering populations (i.e., from the same habitat patch) with ≥ 7 sampled individuals; (ii) a 3-partition scheme including a southern forest block radiating from the Lama forest (c. 50 km maximal radius), a central forest block radiating from the Mont Kouffé forest (c. 45 km maximal radius), and a central forest block in Togo radiating from the Assoukoko forest (c. 25 km maximal radius); and (iii) a 3-partition scheme using a latidunal-based grouping in Benin (South, lower Centre, and upper Centre). Because the first partition scheme yielded the highest levels of differentiation among populations (see “Results”), we calculated their inbreeding coefficients (*F*_*IS*_) in FSTAT.

We used STRUCTURE 2.3.4 [37] to conduct a clustering analysis on all the DGL individuals. We performed 20 independent runs for K = 1–10 using 10^5^ Markov chain Monte Carlo (MCMC) iterations and burnin = 10^4^, assuming admixture model and correlated allele frequencies. STRUCTURE HARVESTER 0.6.94 [38] was used to detect the most likely number of populations (K) using the ɅK method [39]. We also ran STRUCTURE using the LOCPRIOR model (and same other parameters) to assess the amount of information carried by the geographic distribution of populations (r). For r values > 1, the geographic information is considered uninformative [37].

We also inferred the number of populations, spatial locations of genetic discontinuities, and population membership among georeferenced individuals using the *Geneland* package [40] in R 4.0.5 (*R Team Development Core 2021*). Following Coulon et al. (2006), we first allowed K to vary from 1 to 10 and launched five runs of 5 × 10^5^ MCMC iterations (500 thinning and 500 burn-in) under a frequence-correlated model and 1 km of uncertainty for spatial coordinates. Second, we fixed the number of estimated populations on the basis of the first analysis (K = 6–7), to perform 20 independant runs using the same parameters. We also performed 20 independent runs fixing K = 3 as obtained from STRUCTURE (see “Results”). For both analyses, we assessed how stable the population assignment was for individuals among the best (i.e. with highest posterior probabilities) five runs. We used 500 × 500 pixels to map the posterior probabilities of population assignment.

Given the absence of any clear genetic structuring from the above analyses (see “Results”), we performed a spatial Principal Component Analysis (sPCA) using the Delaunay triangulation connection network, which defines neighbouring entities based on pairwise geographic distances. The sPCA is a spatially explicit multivariate approach capable of investigating complex and cryptic spatial patterns of genetic variability from allelic frequencies [41]. Such an approach does not require data to meet Hardy–Weinberg or linkage equilibrium among loci. The sPCA was run with the DGL georeferenced individuals using the *adegenet* package [41] in R, with 9,999 MCMC resampling to infer global *vs*. local structuration levels. Threshold distance between any two neighbors was set to zero.

We tested isolation-by-distance (IBD) among (i) forest individuals and (ii) populations (6-partition scheme; see above) by running a Mantel test in the R package *pegas* [42], where we quantified the correlation (*r*) between genetic (Edward’s) and geographic (Euclidean) distances through 10,000 permutations. The geographic distances among populations were calculated from the center of each forest.

#### Ability of the microsatellite data to trace the pangolin trade

The discriminating power of our microsatellite markers among market and non-market individuals was evaluated by: (i) counting the number of identical genotypes among samples with the Multilocus tagging option in GenAlEx (suboption Matches), (ii) computing the probability of encoutering the same genotype more than once by chance using the R package *poppr* (method = single; [43]), and (iii) calculating values of unbiased probability of identity and probability of identity among siblings (uPI and PIsibs) in Gimlet 1.3.3 [44].

We used the generalized rarefaction approach implemented in ADZE [45] to compute private allele frequencies among various combinations of populations. We delineated six original forest populations after the partition scheme (i), and let population number vary from 2 to 6 (sample size rarefied from 2 to 7). Because the original scheme of six populations yielded the greatest frequencies of inferred private alleles (see “Results”), we graphed the rarefaction curves of each locus for the maximum sample size in these six populations to assess their respective contributions in the identification of private alleles per population. These six original forest populations represent the reference populations that we will use to assign the individuals sampled from wildlife markets. Loci that reached a plateau or showed an exponential trend in their estimated private allele frequencies (selection thresholds were fixed to ≥ 50 and ≥ 45% of privale allele frequency, respectively) were considered as potentially useful for tracing the origin of pangolins found in the markets, whereas loci showing a decreasing trend were discarded. We then crossed these results with the private alleles actually observed for the six populations (GenAlEx), and only considered the loci that showed both observed private alleles (GenAlEx output) and a high potential for tracing (ADZE output). Finally, we manually screened the genotypes of the market individuals to retrieve said private alleles and attribute them to source populations.

#### Demographic history

We tested for bottleneck events in the DGL using the Single Mutation Model (SMM) and the Two Phase Model (TPM) in BOTTLENECK 1.2.02 [46]. We applied the Wilcoxon sign-rank test to analyze the presence of heterozygote excess/deficit using 10,000 replications.

Demographic history was also assessed through the R package *varEff* [47], an approximate-likelihood method that infers temporal changes in effective population size. Given the lack of data on sexual maturity in WBP, we fixed a conservative generation time of 2 years based on estimates from Asian species [48, 49]. Mutation rate was fixed to 5 × 10^–4^ based on published average mutation rate [50]. The analysis was run with the single mutation, geometric mutation and two phases mutation models, using 10,000 MCMC batches with a length of one thinned every 100 batches and JMAX = 3. The first 10,000 batches were discarded as part of the burn-in period. Confidence intervals for ancestral and current effective population size estimates were calculated from the harmonic means for each mutation model.

## Results

### Mitochondrial DNA

**Fig. 1:**
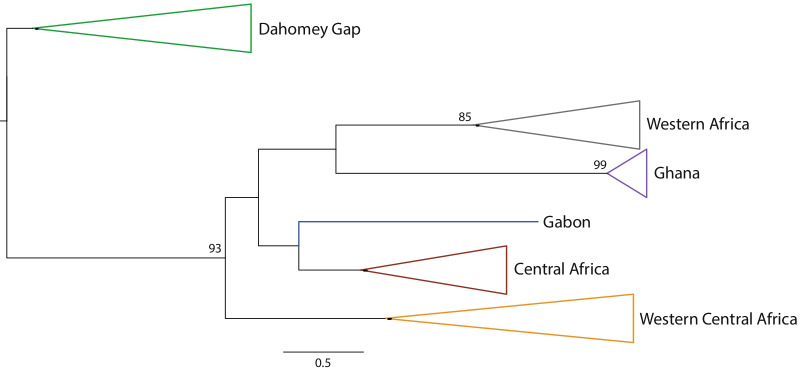
Neighbor-joining tree of white-bellied pangolins based on 268 control region sequences, showing the six main geographic lineages (following Gaubert et al. [9]) collapsed. Bootstrap supports are given at nodes. All the individuals from the Dahomey Gap belong to the Dahomey Gap lineage (see Additional file 3: Fig. S8, for the expanded tree)

Our ML tree based on 268 mitochondrial DNA (mtDNA) sequences recovered the six WBP geographic lineages with robust nodal support, including Western Africa, Ghana, Dahomey Gap, Western Central Africa, Gabon and Central Africa (Fig. 1). All the sequences produced from Togo, Benin and southwestern Nigeria clustered into the Dahomey Gap lineage (bootstrap support = 75%).

**Fig. 2:**
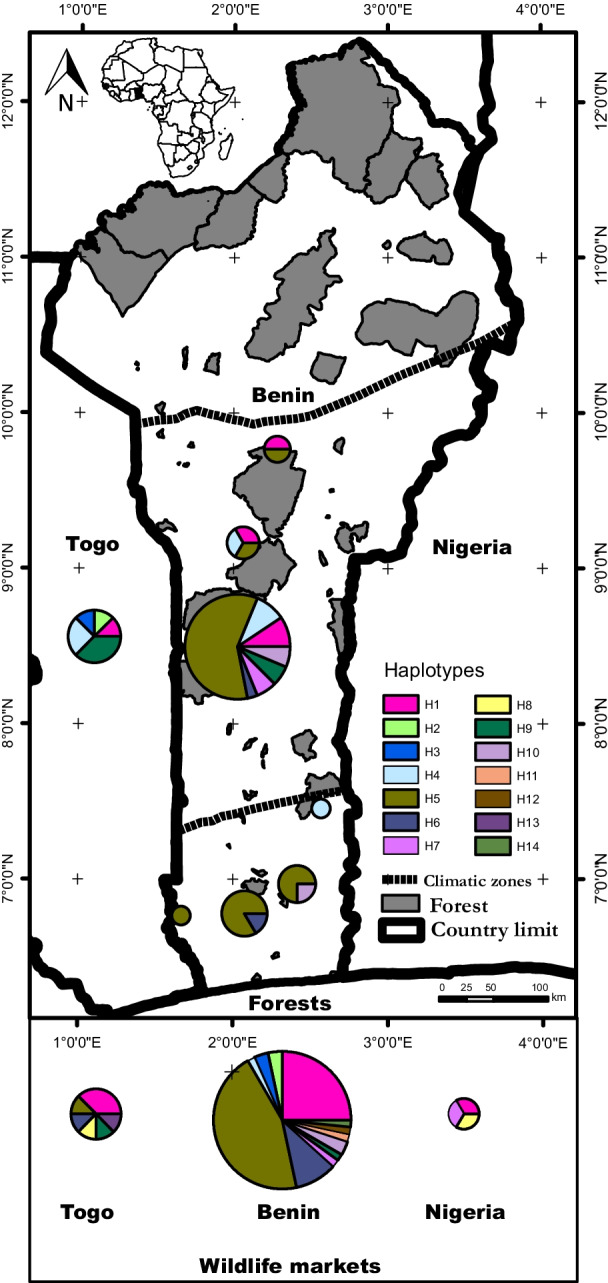
Distribution of control region haplotypes in white-bellied pangolins from the forests and wildlife markets of the Dahomey Gap. Haplotype numbers refer to Additional file 2: Table S2. Top left shows the location of the study zone in Africa (in black)

We identified fourteen CR1 haplotypes in the DGL. The median-joining network did not show any specific geographic structure in haplotype distribution (Additional file 2: Fig. S1). Two haplotypes were dominant in the DGL, with H5 (45%) being widely distributed and H1 (25%) located in the central and northern parts of the range (Fig. 2). Nine haplotypes were found in both forests and wildlife markets, while four were only found in wildlife markets. The proportion of H5 (40%) and H1 (27%) in wildlife markets was reflective of their frequencies observed in forest populations.

Mismatch analysis of CR1 haplotypes showed a bimodal distribution significantly deviating from the sudden expansion demographic model (P(Sim. Rag. ≥  Obs. Rag.) = 0.005), whereas the spatial expansion model could not be rejected (P(Sim. Rag. ≥ Obs. Rag.) = 0.15). The statistics D (− 0.56496), *r* (0.2057) and R2 (0.0710) showed no significant deviation from a scenario of large and constant population size through time (*p* > 0.10), whereas Fs (− 4.780, *p* = 0.0431) significantly rejected the model.

#### Microsatellites

Within the DGL, the number of alleles (Na) varied from 2 to 11 (mean = 5.3). Allelic richness (A_R_) ranged from 1.86 to 8.04 (mean = 4.27; sample size = 169), and observed heterozygosity (*Ho*) and expected heterozygosity (*He*) from 0.072 to 0.775 (mean = 0.414) and 0.069 to 0.842 (mean = 0.498), respectively. Eleven loci deviated significantly from *HWE* (*P* < 0.05). Six of them showed significant levels of heterozygote deficiency (*P* < 0.0025) and three of them were involved in LD (PT_839522, PT_1453906 and PT_353755). Null alleles were identified in ten loci, including seven that deviated from *HWE* (Table 1).

**Table 1:** Genetic diversity estimates at the 20 microsatellite loci used in this study

Locus	*N*	*Na*	*A*_*R*_	*Ho*	*He*	*p(HWE)*	*F*_*IS*_
PT_1162028	169	4	3.123	0.426	0.6	0.000***	0.292*
PT_1753627	162	11	7.993	0.642	0.689	0.019*	0.071
PT_796077	168	3	2.117	0.16	0.186	0.281	0.145
PT_1973508	169	5	4.339	0.562	0.48	0.028*	− 0.167
PT_839522	168	7	6.168	0.524	0.788	0.000***	0.338*
PT_464918	168	8	7.025	0.56	0.766	0.000***	0.272*
PT_1453906	168	4	3.851	0.321	0.646	0.000***	0.505*
PT_34432	151	2	1.863	0.086	0.082	0.580	-0.042
PT_1594892	151	3	2.988	0.192	0.501	0.000***	0.619*
PT_308752	169	11	7.203	0.657	0.678	0.000***	0.034
PT_1669238	169	3	2.233	0.314	0.361	0.276	0.134
PT_1225378	167	3	1.903	0.072	0.069	0.972	− 0.031
PT_739516	167	4	3.363	0.461	0.498	0.612	0.077
PT_619913	168	2	1.959	0.113	0.128	0.142	0.116
PT_338821	165	7	5.930	0.503	0.721	0.000***	0.305*
PT_18497228	168	4	3.848	0.595	0.6	0.611	0.01
PT_378852	168	2	1.969	0.113	0.138	0.020*	0.182
PT_353755	160	9	8.044	0.775	0.842	0.000*	0.083
PT_276641	166	5	4.168	0.699	0.696	0.535	− 0.002
PT_2019332	169	8	5.319	0.503	0.498	0.309	− 0.007

*N* number of genotyped individuals, *Na* number of alleles, *A*_*R*_ allelic richness, *H*_*O*_ observed heterozygosity, *H*_*E*_ expected heterozygosity, *P(HWE)* *P*-value for deviation from the null hypothesis of Hardy–Weinberg equilibrum, *F*_*IS*_ inbreeding coefficient

*P*-values *< 0.05, ***< 0.001

**Fig. 3:**
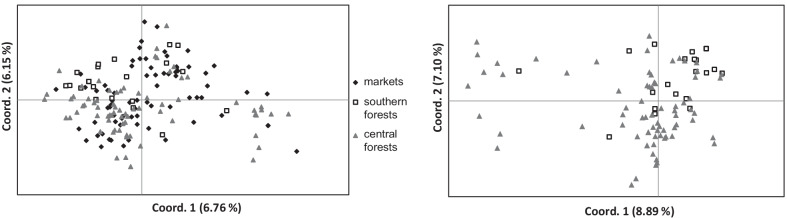
Distribution of genetic variance (PCoA) within white-bellied pangolins from the Dahomey Gap lineage. **A** Including forests and wildlife markets; **B** excluding wildlife markets

Genetic variance within the DGL individuals did not show any geographic structuring on the main PCoA axes (PC1 to PC3 = 12.36% of total variance), whether individuals from wildlife markets were considered or not (Fig. 3).

Paiwise population differentiations using partition schemes (ii) and (iii) ranged from low (*F*_*ST*_ = 0.0378) to moderate (*F*_*ST*_ = 0.116; between central Togo and southern Benin). In partition scheme (i), differentiations among the six forest populations were the greatest, all significant (*p* < 0.05), and ranged from low/moderate (*F*_*ST*_ = 0.0528–0.1399; 67% of the *F*_*ST*_ values) to high (0.166 < *F*_*ST*_ < 0.244; 27% of the *F*_*ST*_ values) (Additional file 2: Table S3). The mean inbreeding coefficient (*F*_*IS*_) in the six DGL populations was 0.172 and varied from 0.098 to 0.317 (Additional file 2: Table S4).

Bayesian clustering analysis with STRUCTURE—without prior information on locations—detected K = 3 most likely number of populations (Additional file 2: Fig. S2). The three clusters did not correspond to exclusive geographic delineations, each being an admixture of individuals from southern and central forest regions together with wildlife markets. The assignment probabilities of the individuals to their respective populations were generally low. Under the LOCPRIOR model, r was equal to 2.368.

Final inference of population number using *Geneland* was K = 7 (95% of the runs). The geographic delineation of the populations did not show any spatially exclusive distribution. Assignment probabilities to populations varied greatly among the best five runs (data not shown). Similar results were observed when fixing K = 3 (Additional file 2: Fig. S4).

**Fig. 4:**
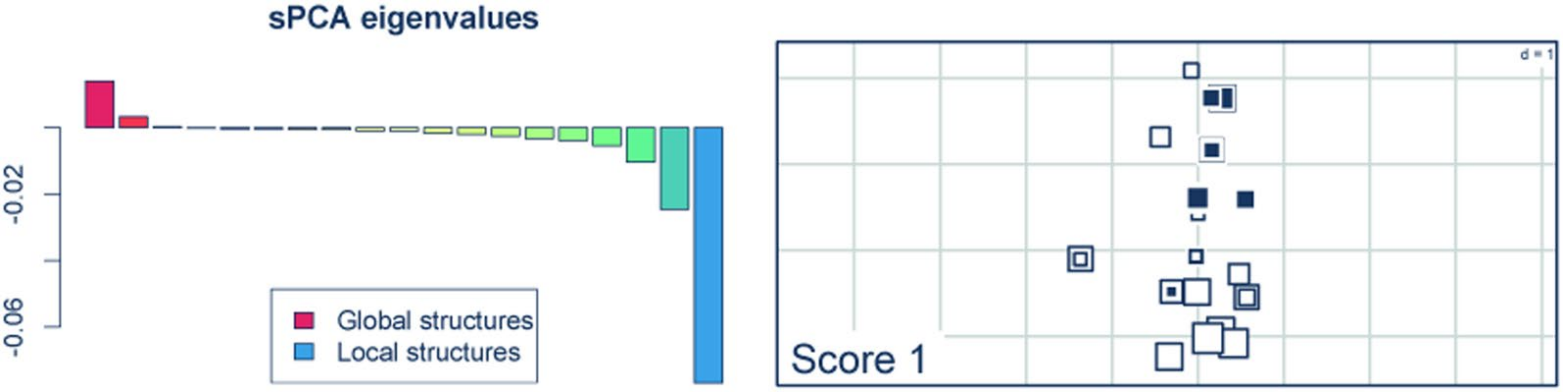
Spatial clustering in white-bellied pangolins from the Dahomey Gap obtained from spatial Principal Component Analysis (sPCA). Left: positive and negative sPCA eigenvalues indicating global and local structures, respectively. Right: Map of the first global sPCA score among sampling localities. Large white and black squares stand for highly negative and positive scores respectively. Large white squares are genetically well differentiated from large black squares, while small squares are less differentiated

The eigenvalues observed from the spCA analysis (Fig. 4) suggested a relatively strong signal of “local structure”, indicating negative autocorrelation between geographic and genetic distances in pangolins from the Dahomey Gap. However, we could not detect any significant global or local structure signal (*p* = 0.602 and 0.102, respectively) across the study area.

**Fig. 5:**
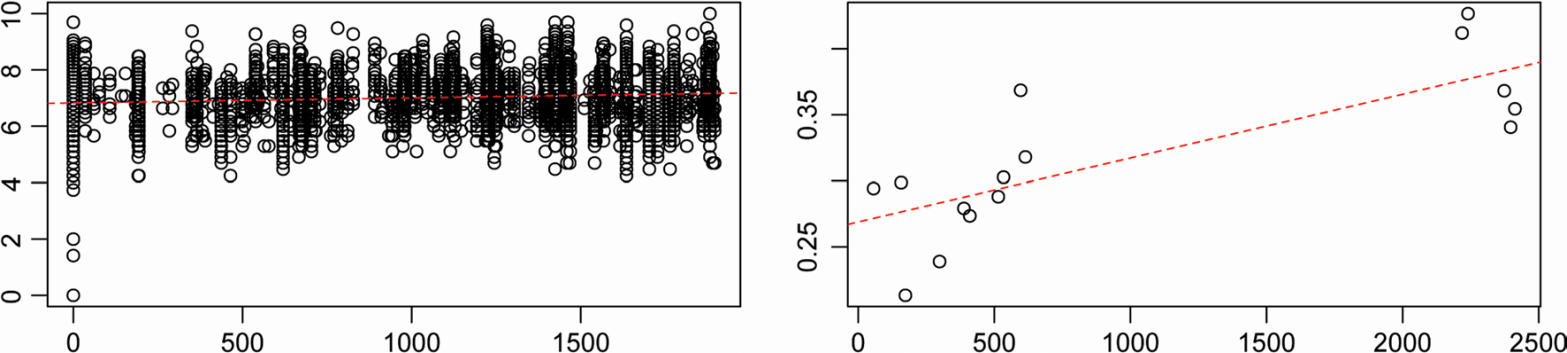
Isolation by distance among (left) individuals and (right) populations of white-bellied pangolins from the Dahomey Gap as inferred from 20 microsatellite loci. Dashed curve indicates linear regression

There was significant IBD effect among forest individuals (*r* = 0.128; *p* = 0.001) and between populations (forests with at least 7 individuals) (*r* = 0.779; *p* = 0.009) (Fig. 5).

A total of 164 samples (97%) had a unique genotype. Five samples shared a genotype with four other samples, including three pairs (A65–A66, B1–B12, B2–B15) and one triplet (D14–D15–D16; see Additional file 1: Table S1). The null hypothesis of encoutering the different genotypes more than once by chance was rejected in all cases (P < 0.0001). The unbiased probability of identity (uPI) and the probability of identity among siblings (PIsibs) were both low (uPI = 8.12 e^−13^; PIsibs = 9.22 e^−06^). At least seven microsatellite loci were needed to reach the conservative value of PIsibs < 0.01 (Additional file 2: Fig. S5).

Estimated mean frequencies of private alleles across the 20 loci (sample size = 7) using ADZE ranged from 0.10 to 0.24. Within the 11 loci that presented appropriate private allele signatures for one or several populations (see Additional file 2: Figs. S6 and S7), seven loci provided six observed private alleles in GenAlEx that could potentially differentiate among four populations. On this basis, five individuals found on wildlife markets could be traced back to their forests of origin: A2 (Gbèdagba market, Central Benin) and A38 (Azovè market, southern Benin) to the Wari-Maro forest reserve, B50 (Azovè market, southern Benin) to the forests of central Togo, and D13 (Avogbannan market) and D26 (Dantokpa market) to the Ouémé supérieur forest reserve.

The bottleneck analysis on the DGL across 20 loci was not significant (Wilcoxon sign-rank test; *p* > 0.05) for both SMM and TPM models.

**Fig. 6:**
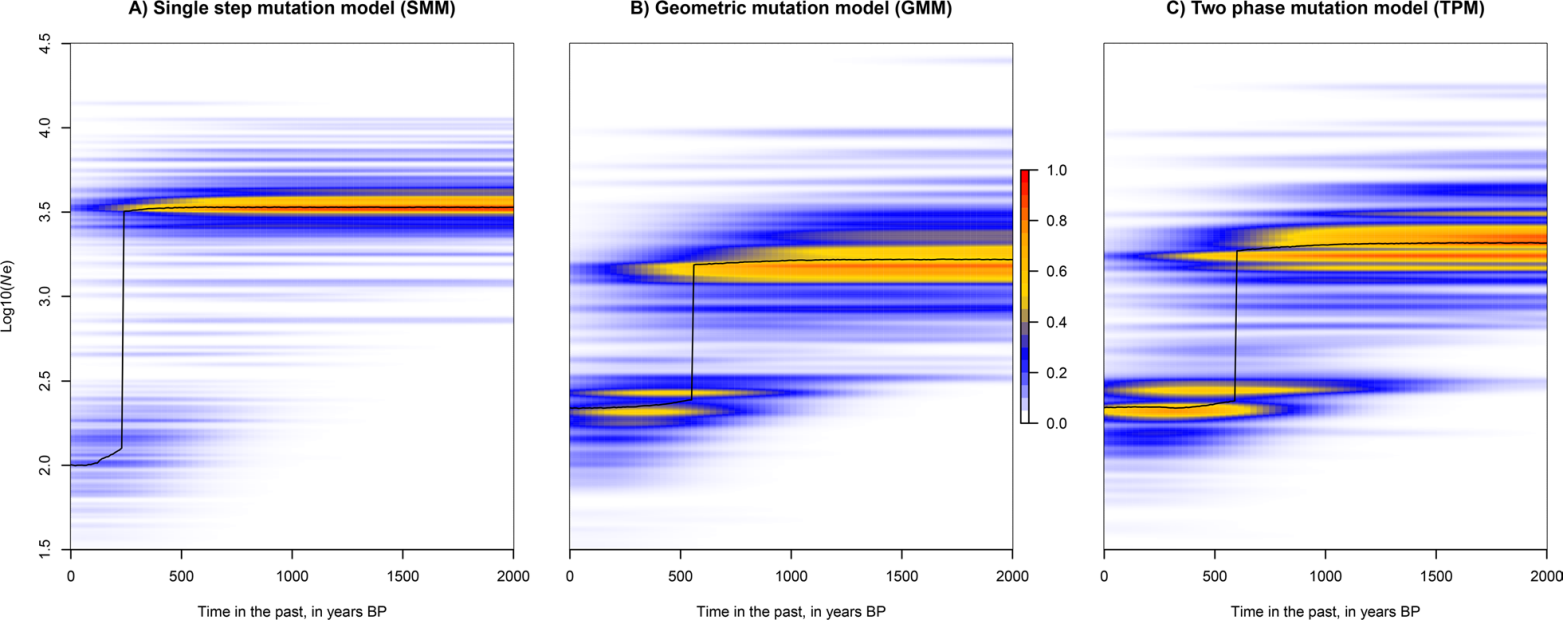
Temporal change in the effective population size of white-bellied pangolins in the Dahomey Gap, as estimated from *VarEff* under three different mutation models*.* Mode (black line) and kernel density (color scale) of effective population size (*Ne*) posterior distributions are given in years BP

*VarEff* identified a pronounced and recent decline in the effective population size (*Ne*) of the DGL regardless of the models (Fig. 6). Our results suggested a 92–98% reduction of *Ne*, from 1682 to 3440 (ancestral *Ne*) to 78–135 (contemporaneous *Ne*) individuals as harmonic means (95% CI 263 to 487). The decrease in *Ne* was estimated to occur c. 200–500 years ago.

## Discussion

### The endemic lineage of white-bellied pangolins from the Dahomey Gap feeds an endemic trade

Our mtDNA tree-based assignment procedure indicated that all the white-bellied pangolins (WBP) collected and traded in the Dahomey Gap—from Togo, Benin and southwestern Nigeria—belong to the Dahomey Gap lineage (DGL; [9]). With 168 new samples sequenced from different forests and TMM, we have more accurately described the geographic delimitation of the DGL, from central Togo to northernmost and southernmost locations in Benin (Ouémé supérieur and Gnanhouizounmè, respectively), and Asejire in southwestern Nigeria. We have also confirmed the absence of range overlap with other WBP lineages, notably from neighboring Ghana. This last result is to be tempered by the fact that introgression between WBP lineages cannot be excluded—but has not been reported to date—and could have passed undetected for the few samples sequenced at a single locus (mtDNA) in our study. The endemic pattern of the DGL superimposes with DNA-based delineation recently found in mammals and plants from the Dahomey Gap [51, 52], further emphasizing the heritage importance of the area for West African forest taxa.

Contrary to the bushmeat markets in Têgon (Benin), Hounkpogon (Benin) and Asejire (southwestern Nigeria) that are known to source the game from nearby forests ([18], this study), the endemicity of the pangolin trade was not expected for the traditional medicine markets (TMMs). This is because the large geographic source from which the TMM network relies was shown in previous investigations from Benin and Nigeria, revealing the long-distance trade of non-native species [53, 54]. Moreover, there is a great demand for pangolins in the Dahomey Gap [55], notably from the Chinese diaspora [7], and the trade of pangolins across borders has been reported elsewhere in tropical Africa [6]. The endemicity of the pangolin trade in the Dahomey Gap might translate into a huge hunting pressure on such a geographically restricted lineage. This is especially true as what we observed in the markets might not encompass the full scale of the pangolin trade, which may also feed the international market through alternative networks [6, 7, 56].

### White-bellied pangolins in the Dahomey Gap show genetic diversity erosion and recent, sharp demographic decline

Overall, the DGL populations were characterized by low levels of genetic diversity. Mitochondrial (CR1) haplotype and nucleotide diversity was lower compared to all the other WBP lineages. Mean allelic richness based on microsatellites was also lower than what was found in WBP from Cameroon using the same markers (*A*_*R*_ = 4.63 vs. 6.74, respectively; minimum sample size = 37; see [17]). Compared to genetic diversity estimates based on ten WBP samples from Ghana, mean observed heterozygosity was again lower in the DGL (*Ho* = 0.541 vs. 0.414, respectively; see [57]).

Low levels of genetic diversity are assumed to be negatively correlated with fitness and adaptability [58]. The overall absence of equilibrium detected from our microsatellite dataset suggests that inbreeding is one of the driving factors of low genetic diversity observed in the DGL (as also observed from the mean inbreeding coefficient among the six populations). Although deviations from Hardy–Weinberg equilibrium (55% of the loci in this study) can be due to a number of factors including inbreeding, population structure, and genotyping errors [59], we can reasonably discard the latter two given (i) the apparent lack of population structure in the DGL and (ii) the optimized loci and genotyping approach that we used. Besides, it is well known that populations going through inbreeding will produce upwardly biased estimates of null allele frequencies [60], as observed in the DGL (50% of the loci). The deficit of heterozygotes observed in 75% of the loci, 30% of which have significant levels of deficiency, also supports the view that the DGL populations are subject to inbreeding, possibly indicative of non-random mating [61].

Our demographic analyses based on microsatellites identified a sharp and recent decline in the effective population size (*Ne*) of the DGL c. 200–500 years ago (100–250 generations), leading to a 92–98% reduction in the current *Ne*. This is below the conservative thresholds of minimum viable population size (500–5000; [62, 63]). As variation in *Ne* is crucial to determining levels of genetic diversity [64], the state of genetic depauperation observed in the DGL may be directly linked to the recent demographic decline affecting the lineage. The time of decline corresponds to a period of major transformations along the ‘Slave Coast’, where from the seventeenth century the Dahomey kingdom expanded as a state bureaucracy benefiting from the growing trade of slaves and agricultural goods with Europeans [65, 66]. Whether such political growth was followed by agricultural expansion and deforestation causing the decline of pangolins in the region is uncertain, but similar declines have been observed in commercially exploited species of vertebrates through the last centuries [66–68]. Because the area underwent drastic alternations of dry and humid periods since at least the last 150,000 year [69], it is possible that the DGL populations were affected by early, successive founder effects and bottlenecks due to Late Pleistocene climatic pejoration [52]. Such demographic events could also have shaped the genetic diversity and absence of population structure (see below) observed today in the DGL. Our results are important for the conservation of the DGL, because inbreeding depression together with high levels of genetic drift will potentially lead to the fixation of mildly deleterious alleles that could drive an extinction vortex in this lineage [70].

Earlier events such as the expansion of agriculture in West Africa c. 4200 BP [71] and natural forest fragmentation caused by cyclical drier climatic conditions in the Dahomey Gap from 4500 BP [10] do not seem to have affected the demographic history of DGL populations. This is due to mtDNA-based demographic analyses showing no deviation from a model of large, constant *Ne* through time, indicating long-term matrilineal stability. The only exception was the Fu’s statistics, which has maximum power to detect sudden demographic decline events [72] and thus can be related to the recent decline discussed above. However, further analyses based on nuclear genomic markers (SNPs) will have to be conducted to assess the ancient demographic history of the DGL, the origin of which dates back to 120–240 kya [9]. The Dahomey Gap is a broad savannah corridor intermixed with forest patches that seperates the two African rainforest blocks, and as such can be considered a sub-optimal habitat for WBP which heavily rely on rainforest cover [9].

### The fragmented populations of white-bellied pangolins in the Dahomey Gap show no genetic structure

Our analyses generally suggested that there was no geographic structuring across the Dahomey Gap, against the expectation that habitat fragmentation leads to genetic isolation [73]. The same absence of structure was observed in the Chinese pangolin among four populations from mainland China [74]. One potential explanation to the lack of population structure would involve long-range dispersals. Pangolins have been reported to disperse up to 300 km in four months, with a marked period of mobility for unestablished young individuals through—notably—anthropized areas [74, 75]. However, the dispersal ecology of pangolins remains poorly known, especially in WBP. The species seems to heavily rely on forest cover and old trees for its nocturnal activities [76], exploring its home range up to 1.8 km per night [77]. In the Dahomey Gap, WBP may occur in disturbed habitats including commercial plantations of teaks and palm trees, fallows and farmlands [78]. However, evidence of long-range dispersal is lacking and the general absence of structural connectivity among the remnant forest islands of the Dahomey Gap, especially in Benin (see [79, 80]) does not support such a scenario.

Although we could not find any clear population structure in the DGL, we detected significant levels of differentiation and isolation-by-distance among both individuals and populations. We observed the strongest differentiations between the most distant Togolese and Beninese populations, and some cases of moderate differentiation between geographically close populations (e.g., the contiguous Ouémé supérieur and Wari-Maro protected areas). Genetic differentiation among populations is determined by the interplay between homogenizing processes such as gene flow and differentiating processes including local adaptation, different adaptive responses to shared environmental conditions, and genetic drift [81]. If we posit that, in the case of the DGL, (i) gene flow between populations is not an option (see above), (ii) genetic drift in isolated, inbred populations should have resulted in detectable geographic structure [82], and (iii) environmental conditions are similar across the DGL range (Guineo-Congolian and Sudano-Guinean zones; [83]), then different adaptive responses of populations to a similar environment could be a candidate scenario to explain the level of differentiation observed among populations from the Dahomey Gap. This could notably be expected in the case of populations trapped in sub-optimal environmental conditions and impacted by frequent climatic oscillations (see [9]). However, microsatellite markers generally reflect neutral genetic variation, and should not be affected by signatures of local adaptation [84].

A more plausible scenario would relate to a former, possibly recent, spatial expansion in subdivided populations across the DGL range, as mtDNA did not reject this model. It is possible that forest-restricted DGL populations underwent a spatial expansion following the last recent increase in rainforest cover during the last Interglacial or early Holocene periods [10]. Such event would explain the absence of population structure together with some level of population differentiation as observed in this study, provided that dispersal among populations would have been maintained long enough to counter-balance the effect of genetic drift in isolated populations later induced by drier climatic conditions and anthropogenic pressure in the Dahomey Gap [85]. Overall, our study has the merit to posit a number of hypotheses that could explain the puzzling pattern observed in the population structure of the DGL. Such hypotheses will have to be tested through a demographic scenario-based strategy, preferentially using versatile and powerful genomic resources.

### Tracing the pangolin trade in the Dahomey Gap: specimen dispatch and evidence for long-distance trade

The genetic diversity of WBP sold on the markets was reflective of the overall genetic diversity (haplotype and allelic frequencies) observed in DGL forest populations, suggesting a widespread sourcing of pangolins through the entire Dahomey Gap. Our 20 microsatellite loci provided the necessary power to confidently distinguish among all the DGL indivuals, and only seven microsatellite loci were needed to reach the conservative value of probability of identity < 0.01 [86]. The probability that two individuals drawn at random from a population, including or not including siblings, will have the same genotype was low (but higher than in previous studies [17, 74]). This has important implications for the genetic tracing of the pangolin trade in the Dahomey Gap, as one of the main inputs of the genetic toolkit is its potential for tracing the trade at the individual level [87]. For instance, our markers would be capable of estimating the exact number of individuals from scale seizures, a major challenge that conservationists are currently facing [88]. We also demonstrated that our genotyping approach was useful in tracing the dispatch of the same pangolin sold on the market (one individual detected on two different stalls in Dantokpa) or kept by local communities (scales of two different individuals shared between villagers in Mont Kouffé and Lama forests). Such application is especially relevant for tracing the pangolin trade, which often forms into separate networks specialized in the selling of specific parts (scales, organs, meat), notably in Benin [7].

Given the lack of population structure in the DGL and the negative autocorrelation between geographic and genetic distances (for individuals and populations), classical assignment procedures [89, 90] are hardly usable to trace the pangolin trade in the Dahomey Gap. We have developed a conservative approach combining rarefaction analysis of private allele frequencies in each population with cross-validation from observed data that could partly circumvent that issue. Such method may be applicable to any taxon or lineage without observed genetic structure across its range, notably at the local scale. We identified seven private alleles (from seven loci) that could potentially differentiate among five DGL populations. Five pangolins were traced to their forest of origin using three private alleles, illustrating the long-distance trading routes that feed TMMs in the Dahomey Gap (see [7]). For example, pangolins from the forests of central Togo, Wari-Maro and Ouémé Supérieur were found on the markets of Dantokpa, Gbèdagba, Azovè and Avogbannan, c. 200–300 km away from the source forests.

Despite a relatively fair number of loci (20) and an exhaustive sample set across Benin, we could only trace c. 8% of the WBP genotyped from TMMs. Such performance could be improved with additional geographic sampling, notably from Togo and southwestern Nigeria, and denser sampling of populations, although the ideal standards for reaching confident estimates of allele frequencies when applied to forensic use might be unreachable in our case (100–150 individuals per population; [91]). Future analyses based on bi-allelic markers such as SNPs will have to be considered as they significantly reduce the sample size required for reliable estimates of allele frequency distribution [92].

## Conclusions

Our study is the first to provide a comprehensive population genetic assessment of an African pangolin species/lineage, filling an important knowledge gap for the future conservation of pangolins in Africa [16]. Overall, we showed that the DGL populations suffered from inbreeding, genetic diversity erosion and a drastic decline in effective population size. Given the multi-purpose trade that DGL populations are the target of (this study, [7]), and the observed reduction of the DGL range during the last two decades (at least in Benin, [11]), the conservation status of WBP in the Dahomey Gap should be urgently re-assessed. Conservation measures are to be implemented before the species becomes locally extinct, as it may already be the case for the giant pangolin [11]. Measures should include the reinforcement and application of the national protection status, the creation of dedicated protected areas and forest corridors, campaigns of public awareness, and breeding programs.

## Supplementary Information

**Additional file 1: Table S1.** General database including geographic references and detailed information on sequenced and genotyped individuals. Empty lines indicate unsuccessful sequencing/genotyping. Haplotypes of CR1 sequences used to compute genetic diversity are provided (H1 to H14). Individuals sequenced successfully but not used to compute genetic diversity—because of missing data—are indicated by “yes”

**Additional file 2: Table S2.** Diversity indices calculated from the control region (CR1) among the white-bellied pangolin lineages. **Table S3.** *F*_*ST*_ values (below diagonal) and associated levels of significance (above diagonal) for the different partition schemes (i), (ii) and (iii). South and North in the partition schemes refer to Benin country. **Table S4.** Inbreeding coefficient in white-belled pangolin populations as delineated in the 6-partition scheme (i). **Figure S1.** Haplotype network (control region) within the Dahomey Gap lineage. Haplotype numbers refer to Table S1. Short bars correspond to mutation numbers. **Figure S2.** Assignment plots among the white-bellied pangolins from the Dahomey Gap as assessed with STRUCTURE for K = 2 (top) and K = 3 (bottom). Each individual is represented by a vertical bar. **Figure S3.** Geographic partition schemes used for computing pairwise differentiations (*F*_*ST*_). (i) 6-partition scheme including the forest habitats with at least 7 individuals, (ii) 3-partition scheme among South Benin-Central Benin-Togo, (iii) gradient-based 3-partition scheme within Benin. **Figure S4.** Spatial clustering of white-belled pangolins in the Dahomey Gap obtained using Geneland for K = 7 (Geneland; above) and K = 3 (Structure; below). **Figure S5.** Unbiased probability of identity (uPI) and probability of identity among siblings (PIsibs) for increasing, optimized combinations among the 20 microsatellite markers. **Figure S6.** Mean private allelic richness per locus in white-bellied pangolin populations (6-partition scheme), as estimated from ADZE (N = 7). FGN: Gnanhouizounmè, FL: Lama, FMK: Mont Koufé, FOS: Ouémé Supérieur, FWM: Wari Maro and TG: forests of central Togo. **Figure S7.** Mean private allelic frequencies per population (forest habitat), as estimated by rarefaction from ADZE. FGN: Gnanhouizounmè, FL: Lama, FMK: Mont Koufé, FOS: Ouémé Supérieur, FWM: Wari Maro and TG: forests of central Togo. The names of the three loci cross-validated (ADZE and GenAlEx) and used for the tracing (i.e. private alleles found in market individuals) appear in green. The four loci cross-validated (ADZE and GenAlEx) but not used for the tracing (i.e. private alleles not found in market individuals) appear in blue. The other four loci revealed using rarefaction but not observed in the actual dataset (GenAlEx) appear in orange.

**Additional file 3: Figure S8.** Neighbor joining tree inferred from the control region and including the six lineages of white-bellied pangolins. The tree includes 181 sequences from the Dahomey Gap, 59 sequences from Western Central Africa, 12 sequences from Western Africa, 9 sequences from Central Africa, 3 sequences from Ghana and 1 sequence from Gabon.

## Data Availability

The nucleotide datasets generated and analysed during the current study are available in the Genbank nucleotide database (http://www.ncbi.nlm.nih.gov/genbank/) under accession numbers OK275650–OK275662. The microsatellite datasets generated and analysed during the current study are included in this published article and its Additional files.

## Abbreviations

CR1: Contol Region 1
DG: Dahomey Gap
DGL: Dahomey Gap lineage
DTT: Dithiothreitol
IBD: Isolation by distance
INRAE: Institut National de la Recherche pour l’Alimentation et l’Environnement
MCMC: Markov Chain Monte Carlo
mtDNA: Mitochondrial DNA
PCoA: Principal coordinate analysis
SMM: Single Mutation Model
sPCA: Spatial Principal Component Analysis
TCM: Traditional Chinese Medicine
TMM: Traditional Medicine Market
TPM: Two Phase Model
WBP: White-bellied pangolin
