# Supplementary figures and images for: Can DNA help trace the local trade of pangolins? Conservation genetics of white-bellied pangolins from the Dahomey Gap (West Africa)
Source: BMC Ecol Evol. 2022 Feb 14;22:16. doi: 10.1186/s12862-022-01971-5 (PMC8842964; doi:10.1186/s12862-022-01971-5)

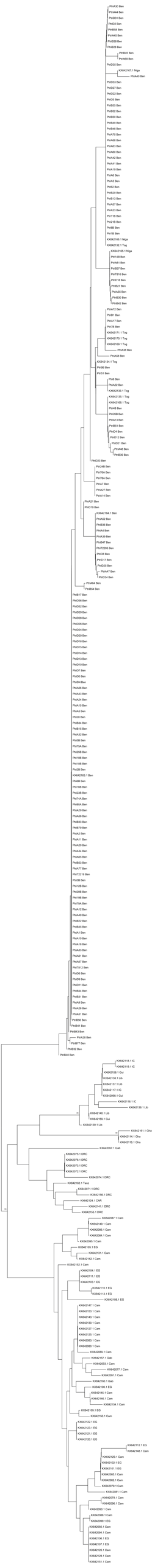

Supplement: Supplementary file 3 — Additional file 3: Figure S8. Neighbor joining tree inferred from the control region and including the six lineages of white-bellied pangolins. The tree includes 181 sequences from the Dahomey Gap, 59 sequences from Western Central Africa, 12 sequences from Western Africa, 9 sequences from Central Africa, 3 sequences from Ghana and 1 sequence from Gabon. [file 12862_2022_1971_MOESM3_ESM.pdf]
